# Supplementary figures and images for: Diospyros rhodocalyx Kurz induces mitochondrial-mediated apoptosis via BAX, Bcl-2, and caspase-3 pathways in LNCaP human prostate cancer cell line
Source: PeerJ. 2024 Jul 1;12:e17637. doi: 10.7717/peerj.17637 (PMC11223595; doi:10.7717/peerj.17637)

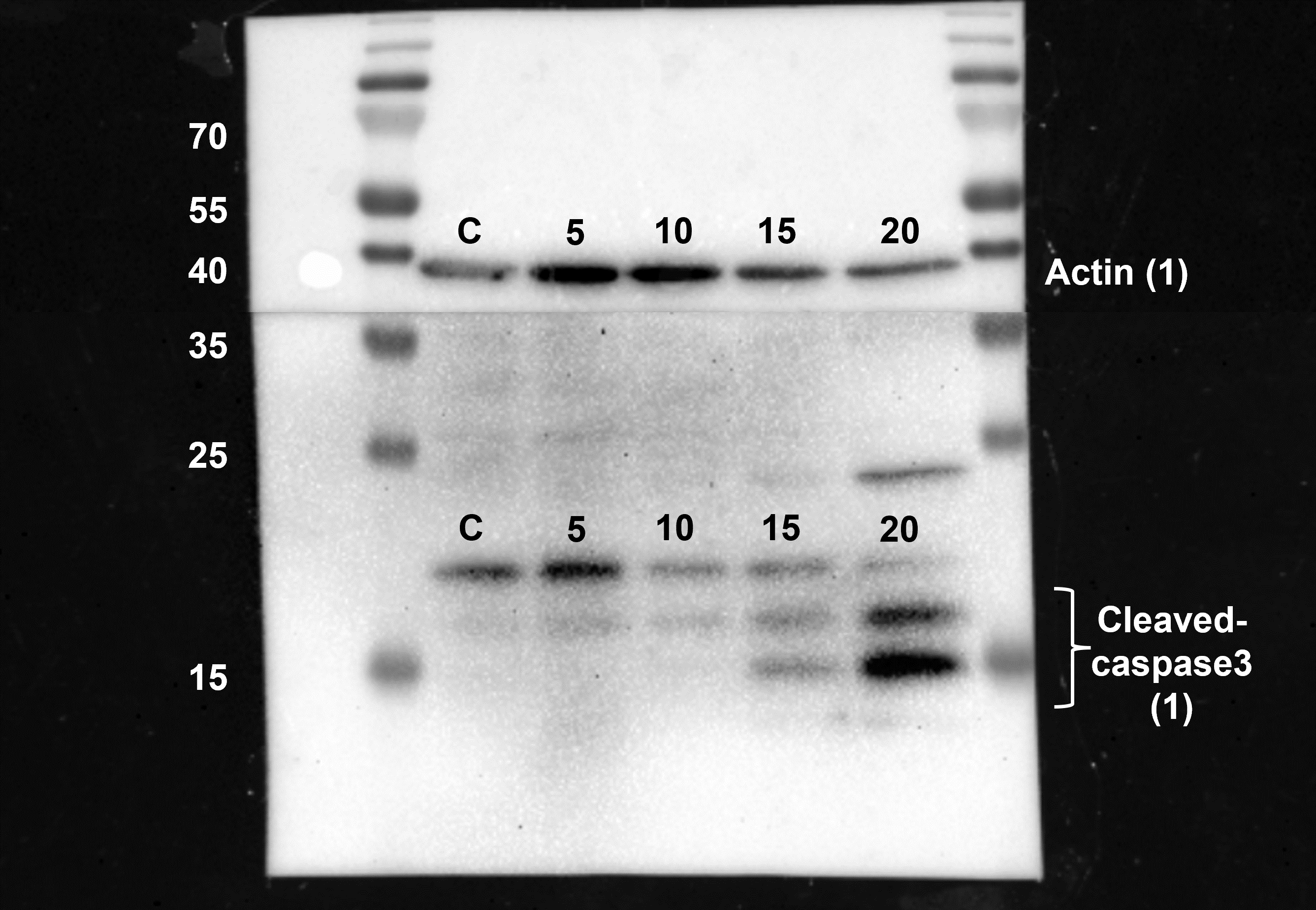

Supplement: Supplemental Information 2 [file peerj-12-17637-s002.zip › Cleaved caspase-3/N1 Cleaved caspase 3 with Actin.png]

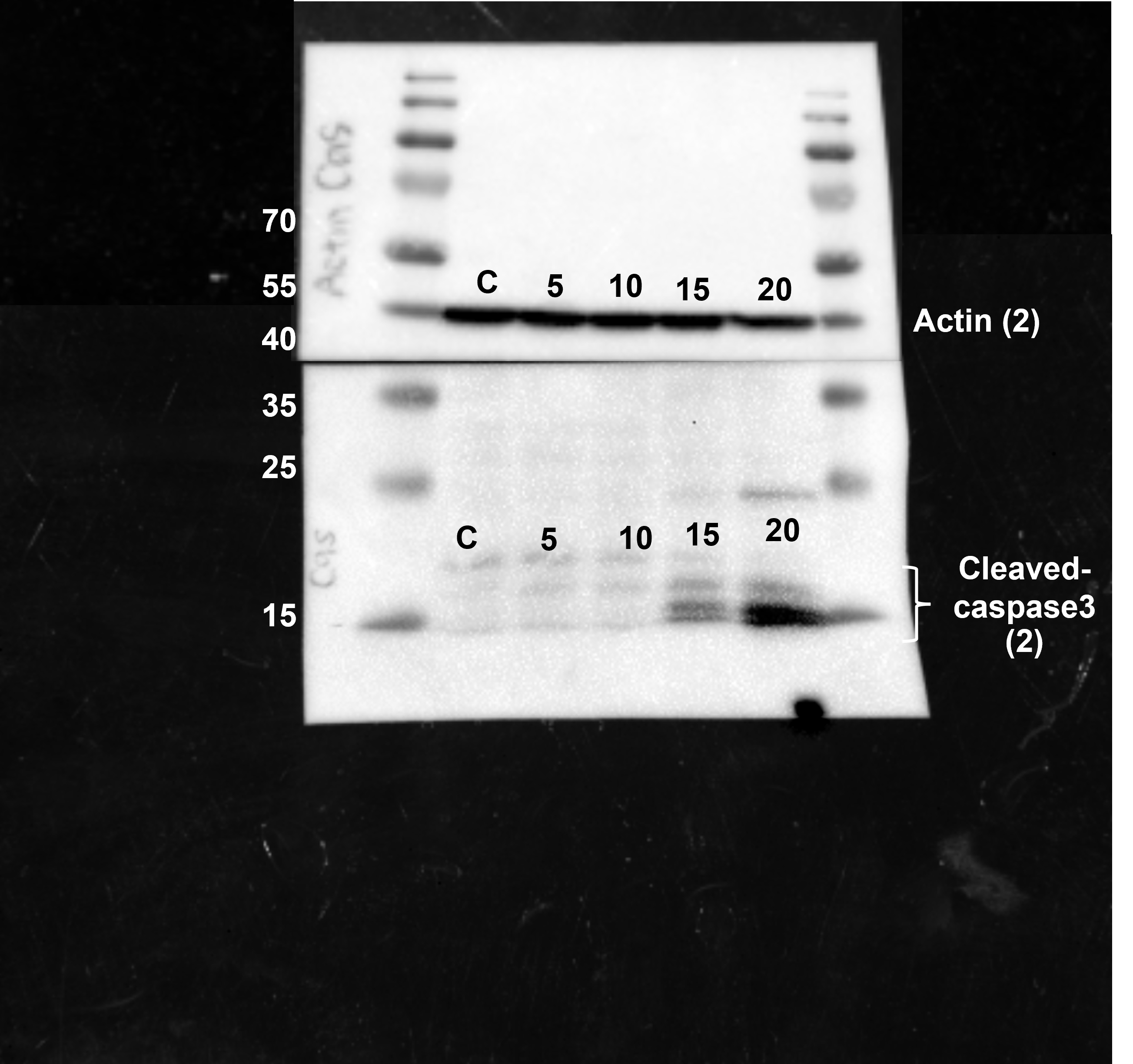

Supplement: Supplemental Information 2 [file peerj-12-17637-s002.zip › Cleaved caspase-3/N2 Cleaved caspase 3 with Actin.png]

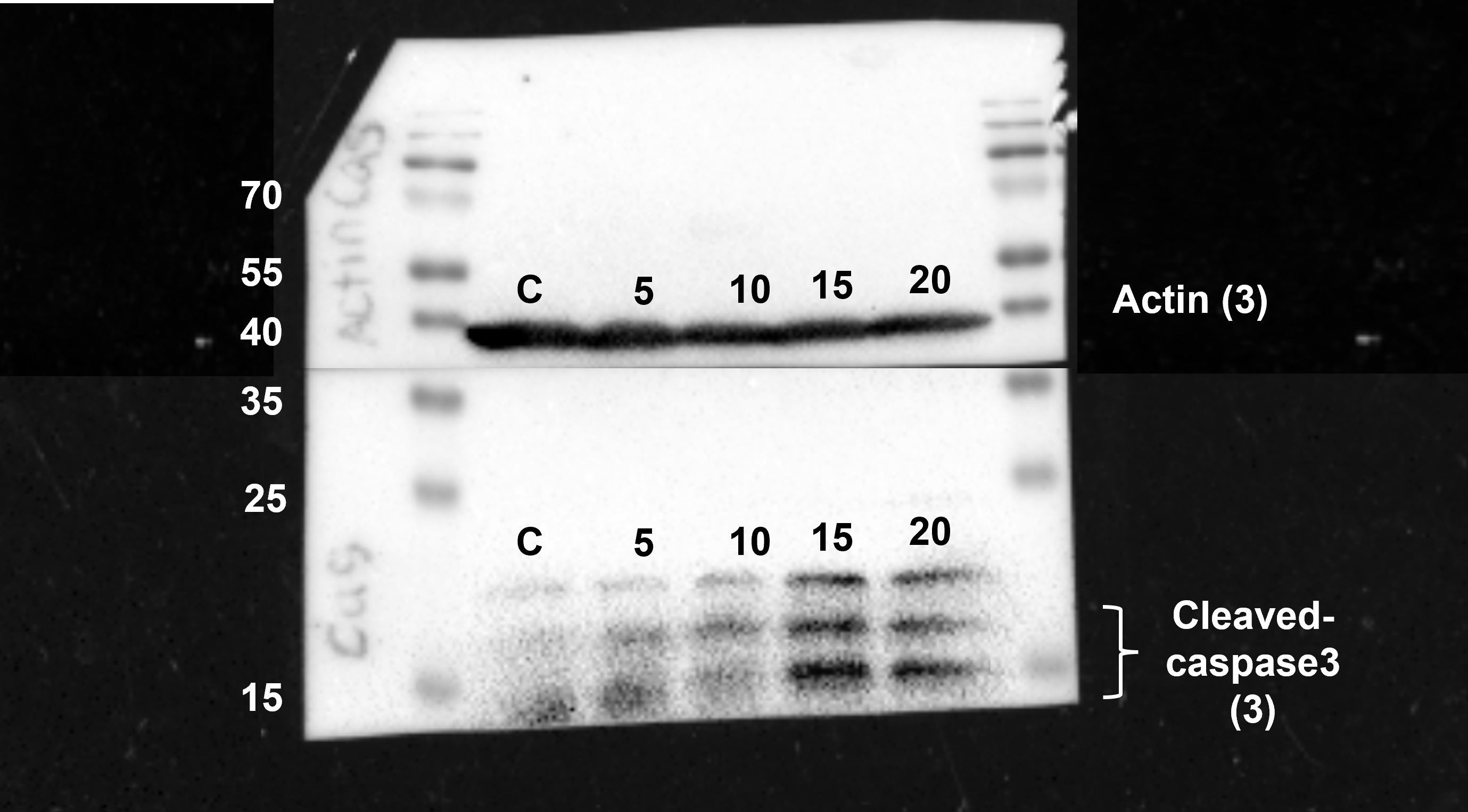

Supplement: Supplemental Information 2 [file peerj-12-17637-s002.zip › Cleaved caspase-3/N3 Cleaved caspase 3 with Actin.png]

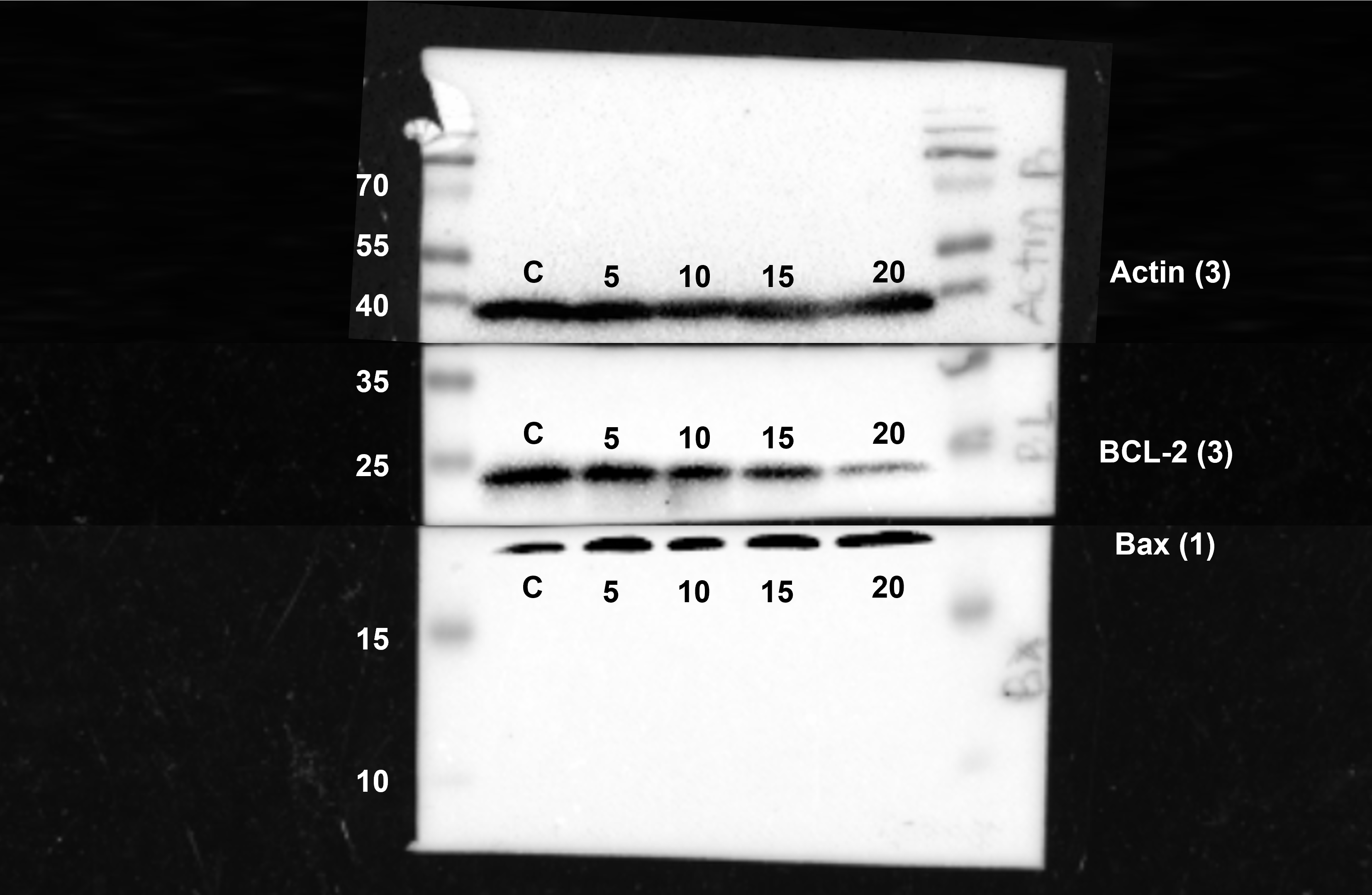

Supplement: Supplemental Information 2 [file peerj-12-17637-s002.zip › BAX/N1 BAX with actin.png]

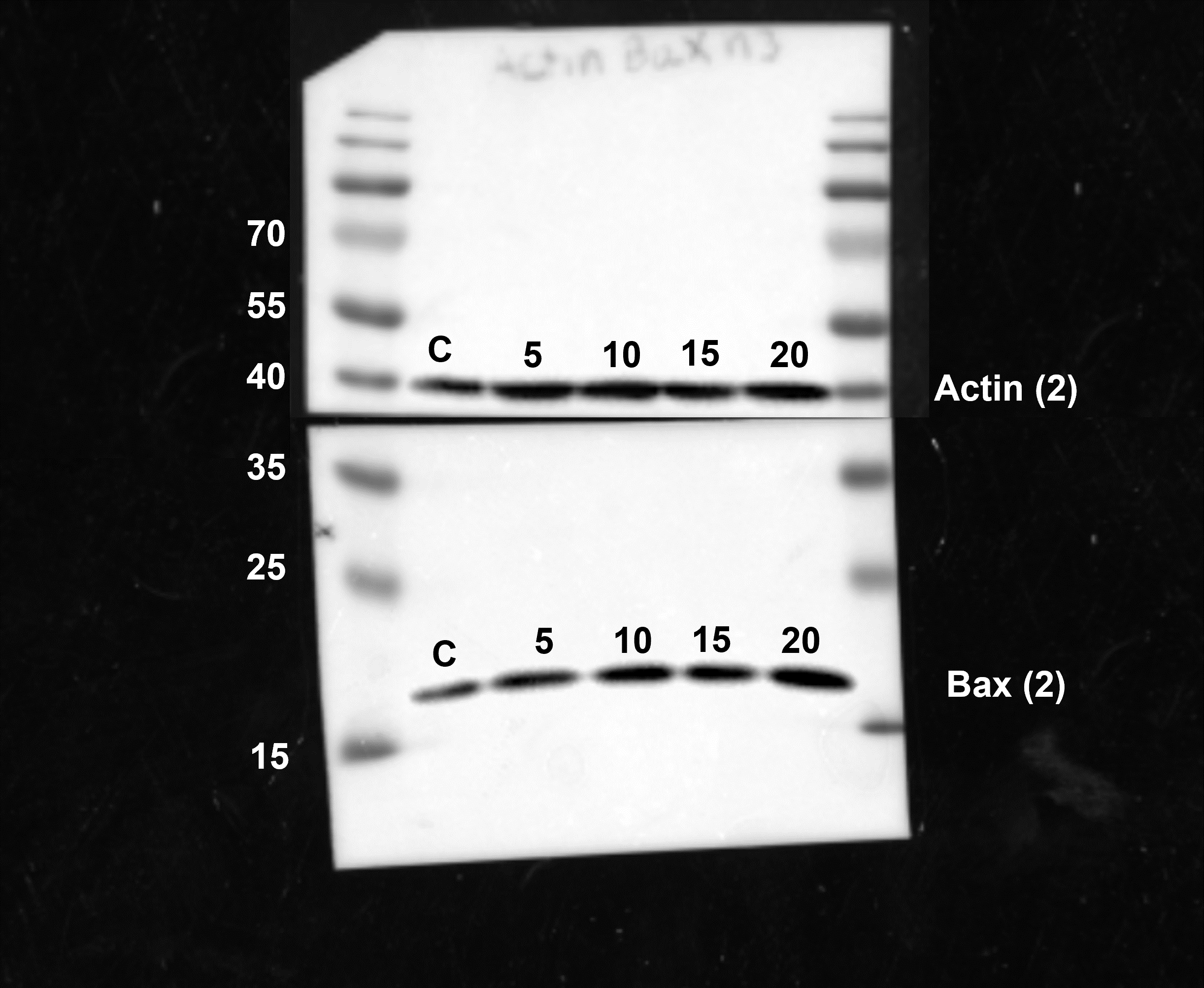

Supplement: Supplemental Information 2 [file peerj-12-17637-s002.zip › BAX/N2 BAX with actin.png]

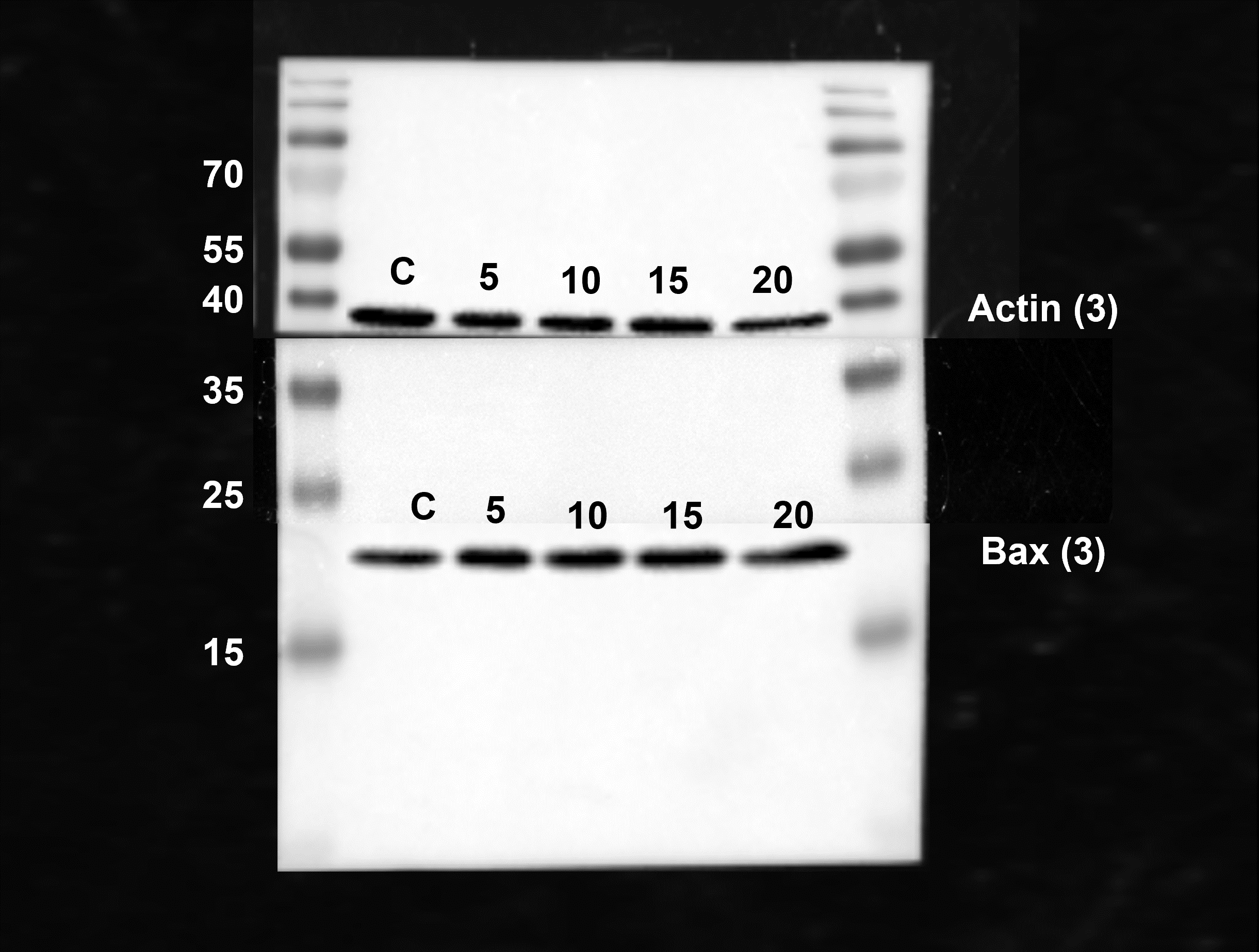

Supplement: Supplemental Information 2 [file peerj-12-17637-s002.zip › BAX/N3 BAX with actin.png]

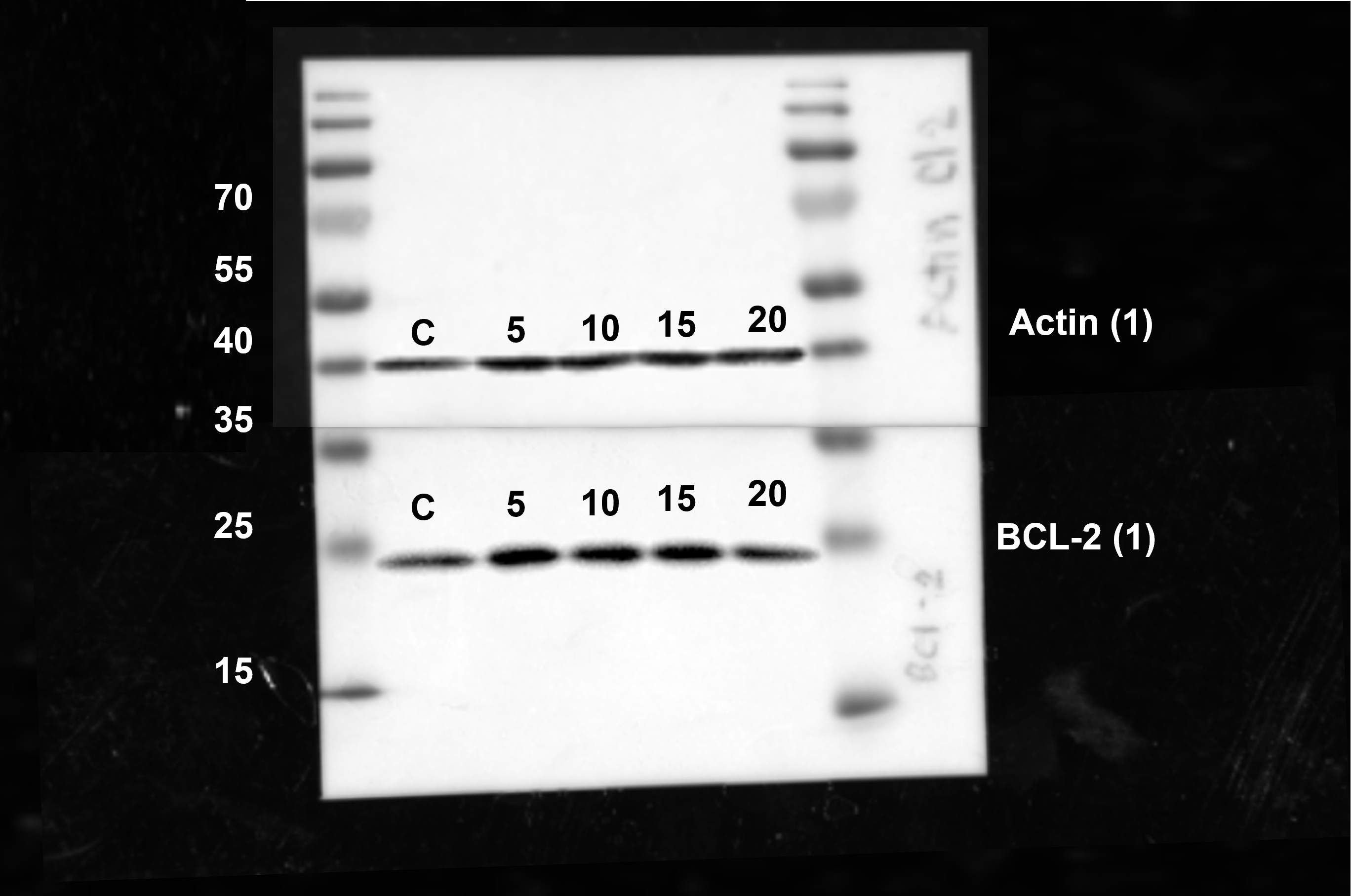

Supplement: Supplemental Information 2 [file peerj-12-17637-s002.zip › Bcl-2/N1 Bcl-2 with actin.png]

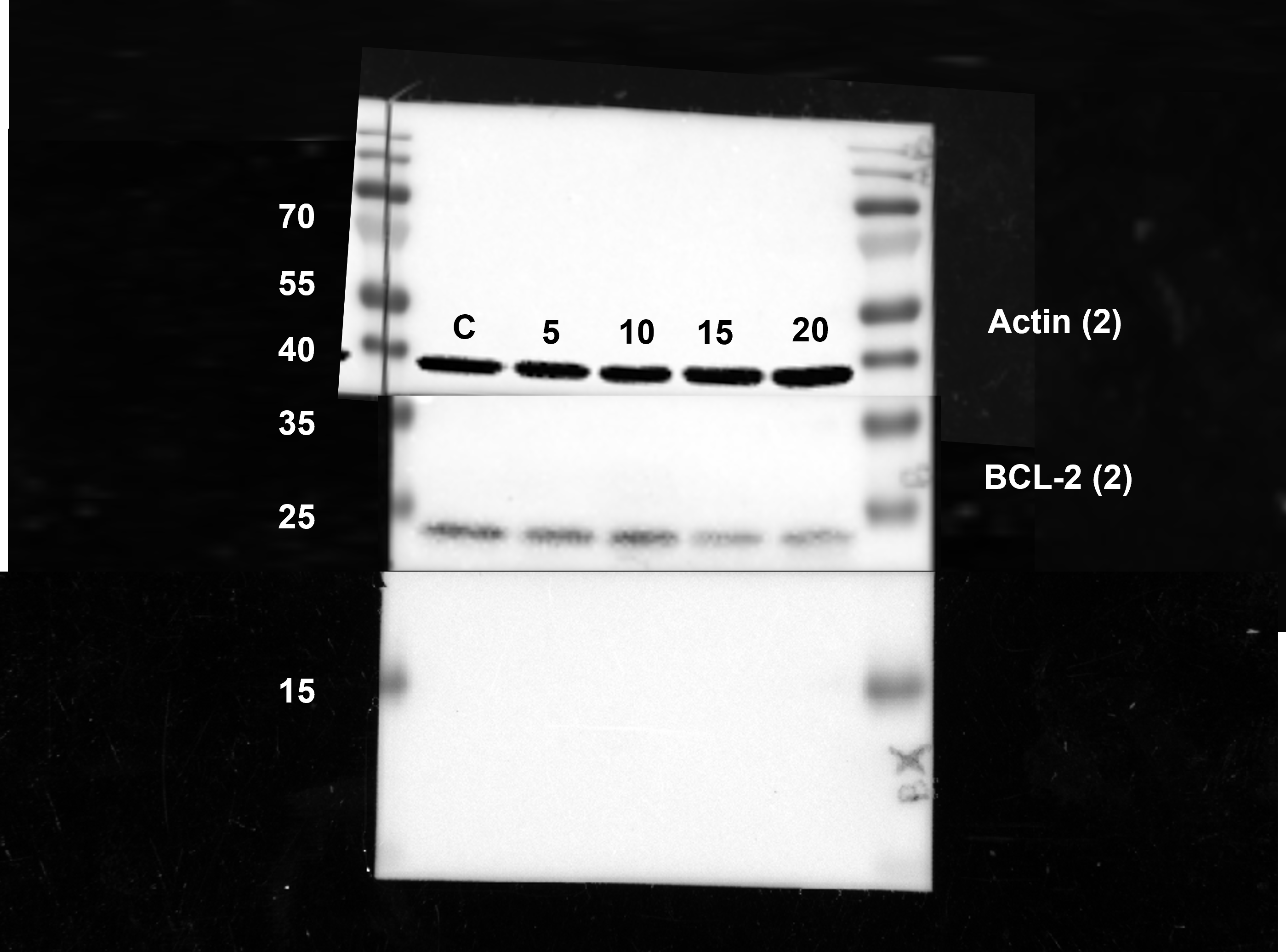

Supplement: Supplemental Information 2 [file peerj-12-17637-s002.zip › Bcl-2/N2 Bcl-2 with actin.png]
